# Surgery Should Complement Endocrine Therapy for Elderly Postmenopausal Women with Hormone Receptor-Positive Early-Stage Breast Cancer

**DOI:** 10.1155/2012/180574

**Published:** 2012-08-27

**Authors:** Olivier Nguyen, Lucas Sideris, Pierre Drolet, Marie-Claude Gagnon, Guy Leblanc, Yves E. Leclerc, Andrew Mitchell, Pierre Dubé

**Affiliations:** ^1^Department of Surgery, Maisonneuve-Rosemont Hospital, Université de Montréal, 5415 boulevard de l'Assomption, Montréal, QC, Canada H1T 2M4; ^2^Department of Anesthesiology, Maisonneuve-Rosemont Hospital, Université de Montréal, 5415 boulevard de l'Assomption, Montréal, QC, Canada H1T 2M4; ^3^Department of Pathology, Maisonneuve-Rosemont Hospital, Université de Montréal, 5415 boulevard de l'Assomption, Montréal, QC, Canada H1T 2M4

## Abstract

*Introduction*. Endocrine therapy (ET) is an integral part of breast cancer (BC) treatment with surgical resection remaining the cornerstone of curative treatment. The objective of this study is to compare the survival of elderly postmenopausal women with hormone receptor-positive early-stage BC treated with ET alone, without radiation or chemotherapy, versus ET plus surgery. *Materials and Methods*. This is a retrospective study based on a prospective database. The medical records of postmenopausal BC patients referred to the surgical oncology service of two hospitals during an 8-year period were reviewed. All patients were to receive ET for a minimum of four months before undergoing any surgery. *Results*. Fifty-one patients were included and divided in two groups, ET alone and ET plus surgery. At last follow-up in exclusive ET patients (*n* = 28), 39% had stable disease or complete response, 22% had progressive disease, of which 18% died of breast cancer, and 39% died of other causes. In surgical patients (*n* = 23), 78% were disease-free, 9% died of recurrent breast cancer, and 13% died of other causes. *Conclusions*. These results suggest that surgical resection is beneficial in this group and should be considered, even for patients previously deemed ineligible for surgery.

## 1. Introduction

Breast cancer (BC) is the most common cancer in North American women. Elderly postmenopausal women are defined as postmenopausal women over the age of 65, or postmenopausal women with comorbidities that render them ineligible for chemotherapy. Elderly postmenopausal women with early-stage BC represent a particular subset of patients because the majority of their tumors are hormone receptor positive (HR+). Blockage of hormone receptor activation with endocrine therapy (ET) reduces cancer cell division and improves disease control. For this reason, ET is an integral part of standard treatment for postmenopausal women with HR+ BC at any stage. ET is used after mastectomy to prevent recurrence, and is also the only therapeutic option in selected stage IV patients. When locally advanced, ET can be used to reduce unresectable disease to a resectable form. As well, it is now often used prior to surgery in early-stage BC to reduce the extent of surgical resection and thus potentially avoid general anesthesia. For many years, tamoxifen was the only ET agent available but studies in the metastatic setting (ATAC, BIG1-98) confirmed the superiority of aromatase inhibitors over tamoxifen in improving disease-free survival [1, 2]. Furthermore, several studies (PO24, IMPACT, and PROACT) found that aromatase inhibitors (anastrozole, letrozole) given preoperatively were as effective as, if not superior to, tamoxifen in improving surgical outcome in this subset of patients [3–5].

The therapeutic challenge for the surgeon is to determine the likelihood of achieving complete response, disease stability, or palliation with available therapies, and to decide whether surgery is indicated. A decision is made according to the clinical and pathological characteristics of the disease, the patient's preferences, the presence of any comorbid conditions, and estimated survival. For example, postmenopausal patients with comorbid conditions may be considered unsafe for surgery, while others may refuse surgery because of concerns regarding perioperative morbidity and mortality, or the need for hospitalization. In such cases, ET represents the only treatment option. 

To our knowledge, no study has yet evaluated ET alone in elderly postmenopausal women with HR+ early BC. ET alone is not widely used in postmenopausal early-stage BC women since it is recognized that a complete clinical response is unlikely. For this reason, most clinicians believe that the cornerstone of curative treatment remains surgical resection followed by adjuvant treatment. However, as discussed above, not all elderly postmenopausal women with HR+ BC will receive surgery. Therefore we devised a study to compare the outcome for elderly postmenopausal women with HR+ early-stage BC treated with ET alone, without radiation therapy or chemotherapy, with that of those treated with ET and breast surgery to determine if surgery could be avoided without affecting survival. 

## 2. Materials and Methods

Medical records collected in a prospective database of elderly postmenopausal BC patients referred to the surgical oncology clinics of one teaching hospital and one community hospital during an 8-year period (January 1, 2000, to December 31, 2007) were reviewed. The diagnosis of BC was confirmed by breast biopsy and the date of diagnosis was taken as the date of biopsy. Elderly postmenopausal women with palpable HR+ (estrogen and/or progesterone receptor expression >10%) tumors, without evidence of metastasis at diagnosis, and who were scheduled to receive at least four months of ET upfront, were included in the study. The reasons to elect for upfront ET were locally advanced disease, the presence of any comorbid condition, and/or the patient's preference. The ET agents used were anastrozole, letrozole, and tamoxifen. Decisions regarding subsequent surgery were taken either at day 0 or after four months of ET using the same criteria. Patients were evaluated every month during the initial four months and every two to three months afterwards. In the case of tumor progression during the initial four months, patients underwent surgery or a change of ET agent. In the case of a partial response, a decision was made based on comorbid conditions and/or patient's preference. When possible, surgery was performed when the maximum best response was attained. 

In accordance with the above criteria, two arms were formed. The study arm was composed of elderly postmenopausal early-stage BC women treated with ET alone, without radiation therapy or chemotherapy. The control arm was composed of elderly postmenopausal early-stage BC women treated with ET and surgery, without chemotherapy. All patients underwent breast conserving surgery. Survival was calculated from the date of diagnosis until death. Two patients in the study arm were lost to followup (at 2.6 and 7.1 months) and were included in the intent-to-treat analysis. Response to treatment was evaluated according to RECIST criteria by measuring the tumor with a caliper. Complete response was defined as disappearance of the tumor on physical examination. Partial response was defined as at least a 30% decrease in the diameter of the lesion, progression as at least a 20% increase in the diameter of the lesion, and stable disease as any tumor size in between. Development of metastasis was considered as progressive disease. Initial response was defined as the response observed at four months, or before if surgery was performed because of progressive nonmetastatic disease.

Demographic and oncologic characteristics were compared between patients who had surgery and those who underwent ET alone. Student's *t* test was used for continuous data while Fisher's exact test was used for all categorical data except the initial tumor's response, which was compared between the two groups with the chi-square test for trend. Log rank test was used to compare survival curves (Kaplan–Meier) between patients who underwent surgery and those who did not. We considered the differences to be significant at *P* ≤ 0.05. Statistical analyses were performed using Prism 4.0 (GraphPad software, La Jolla, CA, USA). 

## 3. Results

Fifty-one patients were included in the study. The study group (ET alone) was composed of 28 women (55%; median age, 86 years; range, 65 to 96 years), and the control group (ET plus surgery) of 23 women (45%; median age, 85 years; range, 65 to 92 years). There was no significant age difference between the two groups (*P* = 0.3145). Patient characteristics are summarized in Table 1. Human epidermal growth factor receptor-2 (HER2) was negative or unknown for the majority of patients since it was not routinely assessed for patients older than 70 years old prior to 2007.

ET in the study arm and control groups consisted of tamoxifen (32% and 17%, resp.), and aromatase inhibitors (68% and 83%, resp.). Results of initial response (0 to 4 months) to ET are summarized in Table 2. There was no statistically significant difference in initial response to ET between both groups (*P* = 0.8781). The median interval between diagnosis and best response was 7.4 months (range, 1.6 to 55.3 months) in the study group, and 3.2 months (range, 1.2 to 7.3 months) in the control group. 

The median interval between diagnosis and surgery in the control group was 6.2 months (range, 2.4 to 11.2 months). 

The median duration of followup was 28.4 months (range, 1.1 to 87.8 months) in the study group, and 63.8 months (range, 37.5 to 109.2 months) in the control group. 

Ten patients (36%) refused surgery at the beginning for personal reasons and remained in the study group, half of which (50%) where still alive at the last follow-up visit. Of these patients, four (14%) had stable disease, two (7%) had progressive disease, of which one (4%) died from recurrent breast cancer 28 months after diagnosis, and four (14%) died from causes unrelated to their cancer. These causes were uterine bleeding (*n* = 1, 4%), cardiac failure (*n* = 1, 4%), and undetermined or not specified (*n* = 2, 7%). 

Two patients (7%) were initially deemed unfit for surgery because of Parkinson's disease (*n* = 1, 4%) and advanced cognitive disorders (*n* = 1, 4%). 

Amongst patients who underwent surgery, none attained a complete pathological response.

At last follow-up visit, in the study group (ET alone), 11 patients (39%) had a stable disease or complete clinical response. Six (22%) had a progressive disease, of which five (18%) died of breast cancer (median survival, 15.8 months). Breast cancer causes of death were distant metastatic disease (*n* = 4, 14%) and contralateral breast cancer (*n* = 1, 4%). Finally, 11 (39%) died from a cause unrelated to their cancer (median survival, 29.5 months). These causes were pneumonia (*n* = 4, 14%), mesenteric ischemia (*n* = 1, 4%), cardiac failure (*n* = 1, 4%), Parkinson's disease (*n* = 1, 4%), infectious colitis (*n* = 1, 4%), and undetermined or not specified (*n* = 3, 11%). 

At the last follow-up visit, in the control group (ET plus surgery), 18 patients (78%) were disease-free, two (9%) died of recurrent breast cancer (median survival, 68.9 months). Breast cancer causes of death were distant metastatic disease (*n* = 1, 4%), and recurrent breast cancer (*n* = 1, 4%). Finally, three patients (13%) died from a cause unrelated to their cancer (median survival, 51.2 months). These causes were a fall (*n* = 1, 4%), and undetermined or not specified (*n* = 2, 9%). 

**Figure 1:**
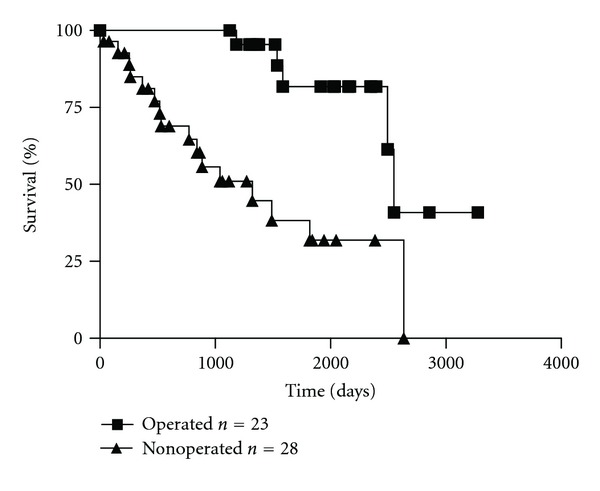
Survival in the study group (endocrine therapy (ET) alone) and in the control group (ET plus surgery), log-rank test: *P* = 0.003.

At five years, the overall survival for the study group was 18%, and for the control group, 52%. The median survival for the study group was 28 months, and for the control group, 64 months. There was a statistically significant difference in survival between both groups (*P* = 0.0003). Survival curves are shown in Figure 1.

## 4. Discussion

This study was devised to compare the outcome for elderly postmenopausal women with HR+ early-stage BC treated with ET alone, without radiation therapy or chemotherapy, with that of those treated with ET and breast surgery, without chemotherapy. The objective was to determine if patients treated with ET alone could avoid surgery without affecting their survival.

Overall, patients in the control group (ET plus surgery) had significant longer survival than patients in the study group (ET alone). A larger proportion of patients in the study group died of comorbid conditions rather than of breast cancer. However, in comparison with the control group, there was still a greater proportion of patients in the study group who died of breast cancer (18% versus 9%). For this reason, we think that surgery is clearly indicated in most cases, even when comorbid conditions are present. Still, the use of ET prior to surgery until the best maximal response has been reached merits consideration. 

The IMPACT trial looked at the effects of neoadjuvant anastrozole, tamoxifen, or a combination of both in postmenopausal HR+ patients with a palpable BC. It concluded that anastrozole is as effective and well tolerated as tamoxifen in these patients [3]. It found no significant differences in the response in patients requiring mastectomy at baseline, but a significant benefit in patients requiring breast-conserving surgery at baseline. The PROACT trial found that neoadjuvant anastrozole is at least as effective as tamoxifen in postmenopausal HR+ BC patients who required breast-conserving surgery at baseline, and more effective in patients who required mastectomy or were deemed inoperable at baseline [4]. Additionally, the PO24 trial showed that neoadjuvant letrozole was more effective than tamoxifen in regards to both tumor response and breast-conserving surgery rates in postmenopausal women who required mastectomy or were deemed inoperable at baseline [5]. In our study, the choice of ET has not been directly studied but letrozole was used most of the time when it became available, initially because of its greater efficacy to reduce estrogen blood levels [6], and then more so after the results of the PO24 study had been revealed [5]. When aromatase inhibitors were contraindicated tamoxifen was used. Anastrozole was used when letrozole was not tolerated; if anastrozole was not tolerated, patients were switched to tamoxifen.

We think that the difference in mortality observed between the study and control groups is due to a selection bias, because patients in the study group had more often severe comorbid conditions and did not receive surgery. On the other hand, patients who had surgery were in better general physical condition. The choice of ET did not appear to play a role.

Randomization of patients could not be performed due to several reasons. First, it is a retrospective study. Second, the decision to operate or not was taken at day 0 or after four months, and was dependent on several factors, including comorbid conditions and patient's preference. For our statistics, use of the multivariable Cox proportional hazards model was considered, but our statistician found it was not feasible.

In this study, the complete response rate was higher in the study group. This can be explained by a longer duration of endocrine treatment, while patients in the control group underwent surgery at some point dictated by a progression of breast cancer or by partial response to the endocrine therapy. A downside to prolonged endocrine treatment is that a proportion of patients may stop responding and eventually progress. On the other hand, continuing treatment beyond the planned four months could have incremental benefits in reducing tumor size and allowing surgery for previously inoperable tumors. This could explain why surgery was performed as late as 337 days after diagnosis. 

## 5. Conclusions

Despite some limitations (sample size, selection bias, no randomization), this study supports the use of upfront ET in most clinical situations in elderly postmenopausal women with HR+ early-stage BC. Appropriate surgery should be performed when the best maximal response has been reached because it reduces BC mortality, even in high-risk patients. At this point, prospective studies should be undertaken.

## Figures and Tables

**Table 1:** Population characteristics.

	Study group “*n*” (range) (%)	Control group “*n*” (range) (%)	*P* value
Number of patients	28	23	
Mean age (year)	84 [65–96]	82 [65–92]	0.3145
Mean initial tumor diameter (cm)	4 [1–10]	4.6 [0–20]	0.2068
ER status > 10%	28 (100)	23 (100)	
PR status > 10%	23 (82)	16 (70)	
Tumor grade I	4 (14)	8 (35)	
Tumor grade II	19 (68)	9 (39)	
Tumor grade III	5 (18)	6 (26)	
Stage I	6 (21)	5 (22)	
Stage II	19 (68)	13 (56)	
Stage III	3 (11)	5 (22)	

**Table 2:** Choice of endocrine therapy and initial response to endocrine therapy.

	Study group “*n*” (%)	Control group “*n*” (%)	*P* value
Anastrozole	2 (7)	6 (26)	
Letrozole	17 (61)	13 (57)	
Tamoxifen	9 (32)	4 (17)	
Complete response	5 (18)	0 (0)	NS
Partial response	10 (36)	17 (74)	NS
Stable disease	11 (39)	3 (13)	NS
Progressive disease	2 (7)	3 (13)	NS

*NS: nonspecific.

## List of Abbreviations

BC: early breast cancer;
ET: endocrine therapy;
HER2: human epidermal growth factor receptor-2;
HR+: hormone receptorpositive.
